# The Relations of Dentistry to Medicine

**Published:** 1879-07

**Authors:** James B. Hodgkin

**Affiliations:** Washington, D. C., Professor of Dental Mechanism and Metallurgy in the Baltimore College of Dental Surgery.


### ARTICLE IV.

### *The Relations of Dentistry to Medicine.*

[An Address read before the Baltimore Medical and Surgical Society, May 7, 1879.]

BY JAMES B. HODGKIN, D. D. S., OF WASHINGTON, D. C.,

Professor of Dental Mechanism and Metallurgy in the Baltimore College of Dental Surgery.

GENTLEMEN:—It has been perhaps a year since I stood before you, in response to your kind invitation, to give you a few thoughts on "Some of the causes of the decay of the teeth of Americans;" and I believe that there was then a sort of tacit understanding that I should supplement the two addresses then made, with some reflections on "*the relations of dentistry to medicine.*" I have felt that this was due, not less to my own, than to your calling, from the fact that our practice so often overlaps, and that we find consultations with you useful. And as nothing so conduces to harmony and good fellowship as a perfect understanding of the legitimate field of operations of fellow-workers in life, I trust that what I may have to say will tend to show that while the field of each specialty in medical practice is more or less well defined, still there is common ground, on which all may meet. And I am quite sure, moreover, whatever may have been the feeling in the past, and whatever the repugnance on the part of the medical professsion to a recognition of the claims of dentistry to a place as a department of surgery, that now there is a growing recognition of the kinship between the two ; and indeed, my presence here to-night, in the character of essayist, is a candid recognition on your part of the claims we may have, and that however narrow the field that we (the dentists) occupy, we have not failed entirely to develop some truths worthy of public recognition; is a proof that you feel that we know something which you wish to hear.

*But is this a fact?* What claim has dentistry to recognition at your hands as a specialty of medicine, and what proof is there in the limits set for it by mutual consent—bounds by tacit agreement—that dentistry has claim to fellowship with those who occupy the broader field of which you are representatives?

Is it a fact that we are at least *cousins*, if not *brothers?* or are we simply clever artisans, skilful mechanicians, worthy of praise, but only deserving of classification with the manufacturers of artificial limbs, of trusses, of glass-eyes, and wigs?

Of late there has risen in the ranks of the dental profession an earnest desire for some more complete recognition at your hands than has been our fortune to have accorded us. There are now not a few among us who have boldly claimed their right to a place among the M. D's., and who have met, in their advances or strivings for advancement, severe rebuffs. They have said that inasmuch as dentistry is a specialty of medicine, that they as specialists were quite as good as the other specialists—the oculists, aurists, &c., and harsh words have been uttered, and recriminations indulged in because the doctors of medicine would not take them by the hand and call them brothers; forgetful of the fact that true merit in the long run is always recognized wherever found, they have rather striven to push themselves. And, on the other hand, I fear that the tendency on the part of the older and more aristocratic profession to "turn the cold shoulder" to our advances, is nearly as marked as the fault—shall I call it presumption ?—on the other hand.

But, I infer that a better state of things now exists, a more generous feeling, on all sides. And I should do the profession of which you are honored members a great injustice did I suppose for an instant that you were intolerant of any one who had contributions, no matter how meagre, to offer —contributions which would tend to mitigate the world's suffering.

I have asked the question whether or not dentistry is anything more than a highly developed mechanical pursuit, and whether those who pursue it can justly claim any higher position than that of clever manipulators. Are we deserving of higher rank than the artificers of wooden legs, &c ?

If the relief of human suffering be any title to admission to the ranks of the medical profession; if the fact that the woes of humanity are mitigated, relieved, cured, is a title to such rank, then certainly the dentist should claim a good deal. For in no department of surgery or medicine are results so certain, operations so successful, relief so abiding, as in dentistry. But I take it that such claims, though fully admitted, hardly entitle us to a place in the world's estimation with you, unless it is shown that such relief as is claimed to be given is the outcome of pathological knowledge. Unless I show that the foundations, on which the superstructure of our surgery and theory is builded, are laid broad and deep in as thorough knowledge of the conditions of the human body in health and disease as is possessed by you, I shall fail to make good the claim which certain of my brethren make to relationship.

And here I candidly confess that so far as my knowledge of my own profession goes, its members are sadly at fault. The fact that our calling is largely mechanical is admitted; and this fact alone is an explanation, in part at least, of the lack of broad culture and of extensive physiological and pathological research. The man whose daily pursuits compel him to manual training rather than to investigation into the causes of things, whose constant occupation consists in handling instruments, and in cultivating touch and tactual dexterity, and whose success depends upon his accomplishments in this direction—such a man, however he may desire a broader, general development, can scarcely attain it, seeing that his daily life constantly draws him away from it. Routine is unfavorable to breadth. Then, too, many who pursue dentistry, elect their calling because of real or fancied talents of hand-craft.

Still, I can but feel that the claim to recognition has its force, if properly viewed. The cry of suffering humanity for relief from pain, how it appeals to every sympathy, how it moves our hearts, and stirs our emotions! The appealing cry for help, for relief from distress and anguish, has been, is, and must be the moving impulse of the healing art. He who came into the world to seek and save lost souls, never forgot for a moment that the souls had bodies (you see, gentlemen, that I am old-fashioned in my beliefs,) and it is hard to decide in reading the story of that wonderful life whether he most pitied the degraded mind and soul, or the degraded and suffering body. The public, who failed utterly to recognize his spiritual mission, gladly availed themselves of his powers as a physician, and in no case did he turn one away. Pitiful and tender, he "bore the griefs and carried the sorrows of many." And so it is that in all ages the noblest type of manhood is that which devotes itself earnestly and heartily to the work of alleviating the sufferings of its fellow-men; and he who does not feel this is unfit for the physician's office and function.

Such office and function is that of the dentist in large degree, and whether the means taken be those of scientific study of cause and effect—the causation of disease and the effect of remedies—or whether by a simple mechanical operation upon the decayed tooth-structure, pain is prevented—in any event, he who either mitigates the sufferings of his fellows or prevents them, is deserving of reward and honor.

While I have alluded to the fact that the peculiarity of the practice of dentistry tends to largely develop the mechanical skill resident in a man, and that this fact of its being largely mechanical in its nature hinders the breadth which might arise, did the relations of cause and effect require closer study on his part, I do not mean to allow myself to be understood as saying or inferring that we have not among us men who can lay claim to the title of "scientists." For I tell you that dental literature exhibits no mean record of things within its scope, and that the investigations of some of those who have been and are now at work in histological research, are worthy of classification with the best efforts of the best men of the day. That for accuracy and breadth of physiological culture, for acuteness of diagnosis, for skilled application of therapeutical means, we have men who are the peers of any in the regular practice of medicine.

But with all this, I am free to say here for my profession, and representing as I do in part the oldest dental college in the world, that the masses of our profession would be little profited by being taken into association with yours. Poorly cultivated, restricted, narrow, with the conceit which so commonly goes hand in hand with ignorance, they are yet a long way from deserving more than the term "clever mechanics;" and this too in spite of colleges and teaching. "Is dentistry a liberal profession?" was the title of a very thoughtful essay by one of the thinkers of that profession, and it suggests much of sadness to me. Ignorant of much, versed in little, clever manipulators, the average dentist is in need of much development. Whether those who come to our colleges as raw material are inferior in point of talent to those who crowd the amphitheatres of your medical schools, I am not in a position to judge; but I hear from those who are judges that ours are quite equal—some say they are superior—to the other. And indeed I see no reason why, in consideration of the fact that such material is drawn from all over the country, there should be a difference in favor of the medical classes. Certainly our students are nothing to boast of. Are yours?

But I only slowly approach the subject, and must recall the fact of the shortness of time.

What have we done as dentists? on what do we base our claims to relationship to you?

*What has dentistry done?* I have not strength to tell, and certainly you have not time to listen to it all. But I may, very briefly, outline some of the work which, within the past fifty years, has been accomplished.

The work of surgery cannot be over-estimated, (the benefits conferred on mankind by the use of drugs is often called in question,) but of *surgery* no one can hesitate to say that the greatest boons conferred on suffering human beings have come through a knowledge of how to relieve pain and arrest disease by operations. It is hard to say how it came to pass that the work of removing aching teeth fell into the hands of persons outside the medical profession; but it is certain that until the last fifty years or less, the barber, the blacksmith, the druggist, were the extractors of teeth. Probably it was due to the fact that this operation was looked upon as purely mechanical, requiring only strength of wrist. And until about the period I mention, the operation in question was the only one called upon to be performed. Since then the work stands about thus: Artificial teeth, rivalling in color, shade, tone, shape, &c., more nearly the work of nature than anything in art, save and except artificial flowers, and taking the place very fairly of the natural organs as agents of mastication, have been brought to a perfection which demands little improvement. The ancient crone, with "nose resting on chin like a staff," the snaggle-tooth disfigured man and woman has disappeared, and youth and beauty have taken the place of decrepitude, in appearance. Teeth are filled, abscesses cured, chronic fistulous discharges through the gums and cheeks healed; exposed and aching pulps of teeth are capped and rendered healthy; teeth extracted and replanted with success; artificial teeth grafted on natural roots—in a word, restoration taking the place of ruin. It is certain that in no department of surgery can success be so certainly predicted as in dentistry.

In accuracy of diagnosis, in skill in therapeutic treatment, in a perfect knowledge of the conditions of disease falling into their hands, and of skilful application of remedies, I am sure that those who are familiar with the practice of the *best* dentists will agree with me that they are the peers in their specialty with the best surgeons, and produce results as beneficial to the human race.

*How much recognition should dentists claim, and what should you be willing to accord them?* I do not think that any dentist should ask for more than this at the hands of the general practitioner; I do think that the intelligent and cultivated dentist is entitled to this much:

(1.) In all lesions of the trifacial, and all diseases arising therefrom, the dentist should be consulted, to see if his special knowledge will avail in making a diagnosis.

(2.) In all carious conditions of the bones of the face, and troubles arising therefrom.

(3.) In nervous diseases, where there is any probability, or even possibility, of involvment of nerve centres through the trifacial.

(4.) In digestive disorders, where even remote suspicion rests upon the teeth.

I do not think that the dentist is called upon, or should be expected to perform large surgical operations, upon the face or mouth, as the removal of maxillæ, amputation of the tongue, staphylorrhaphy, &c., although I am aware that such claims are made by some of my brethren. Nor do I believe that he should, as a rule, prescribe for general diseased conditions. But he should, on the other hand, be able at least to *suspect* troubles which, distantly located, express themselves in facial reflex maladies, and refer his patients to you. I here express the opinion often expressed to my students, that as I feel that you, gentlemen, are trenching on my ground when you attempt the extraction of teeth, so we should be overstepping our limits in playing the role of the general practitioner. But further, I do teach and enforce always the doctrine, and emphasize it with all the earnestness I have, that dentists should *know*, fully and deeply, the fundamental principles of physiology and pathology, and should be able, if occasion demands, to give advice in cases of emergency. The spectacle of the dentist in case of accident or untoward manifestations from an anæsthetic, running off after the doctor of medicine, unable himself to apply even the simpler remedies used in such cases, is to me a disgrace and a shame. In all such emergencies he should be able to meet them, or else discard the agents which render such accidents liable.

Should dentists be required to hold medical diplomas, and should the study of medicine be a condition precedent to the study of the other? I think not, at least at present. The curriculum of a dental school furnishes a dentist with a fair outfit, and we claim to teach all of anatomy, physiology, &c., that is needful. But I do not discourage, but encourage, my young men to study medicine, if only for the broadening effect. Some of the best men in the dental profession have come into it from the medical, and I leave it to you to judge whether they stepped up or down.

Finally, as I earnestly advise my brethren of the dental profession to cultivate the acquaintance of the physicians, so I advise you to cultivate the acquaintance of your dentist. Draw him out, inspire him with zeal, not only to do good work, but to *develop* himself. Let him see that you feel him entitled, as a worker for suffering mortals, to recognition in some degree to brotherhood. Let him feel that you appreciate what he *does* know, and encourage him to larger growth. If he needs developing (and most of us do,) help him. And have your dental mate; if possible, send your patients, not to some dentist, but to *the* dentist whom you endorse, or fraternize with. If you help him, possibly he may help you.

And more, I suggest that you look into dental literature. You may see some things which will surprise you. Original research, comprehensive judgment, acute analysis, superior thinking powers—all may be seen in larger measure than you possibly think. There are no more earnest students anywhere than some of our men, and fifty years of study is not and cannot be fruitless of result. Ten colleges, six or seven journals, &c., annual conventions, State and national, show that earnest men are at work; and earnest men are the need of the times.—*Southern Clinic.*

